# Pennsylvania State Dental Society

**Published:** 1893-08

**Authors:** 


### PENNSYLVANIA STATE DENTAL SOCIETY.

The Pennsylvania State Dental Society held its annual meeting, July 11, 1893, at the Mountain House, Cresson Springs. In the absence of the President the session was called to order at ten o'clock by the Vice-President, Dr. F. L. Bassett, of Philadelphia. Twenty members were present. A letter was received from the President, Dr. W. E. Van Orsdel, stating his inability to be present; also a message from Dr. Louis Jack, stating his absence on account of illness.

Reports from committees and the Treasurer's report were read. These embraced interesting and important communications from the Committee on Enforcement of Dental Law, Examining Board, and on Legislative Action. The officers elected for the ensuing year were, President, Dr. F. L. Bassett, Philadelphia; First Vice-President, Dr. J. A. Libby, Pittsburg; Second Vice-President, Dr. G. W. Green; Treasurer, Dr. H. N. Young, Wilkes-Barre; Secretary, Dr. C. V. Kratzer, Reading; Assistant Secretary, Dr. R. B. Cummings.

*Examining Board* (in place of expiring members).—Dr. Louis Jack and Dr. L. Ashley Faught.

*Committee on Revision of By Laws.*—Drs. Libby and Kratzer.

*Committee on Enforcement of Law.*—Drs. Faught, Hamilton, Fundenberg, Boyce, and Phreaner.

*Executive Committee.*—Drs. Roberts, Young, Litch, Magill, and Klump.

*Publication Committee.*—Drs. Magill, Fundenberg, Green, Keiffer, Kirk, and Kratzer.

*Committee on Legislative Action.*—Drs. Klump, Magill, Libby, Boyce, Young, Thompson, and Peirce.

*Clinical Committee.*—Drs. Filbert, McQuillen, and Cummings.

